# Modulation of TRPV-1 by prostaglandin-E_2_ and bradykinin changes cough sensitivity and autonomic regulation of cardiac rhythm in healthy subjects

**DOI:** 10.1038/s41598-020-72062-y

**Published:** 2020-09-16

**Authors:** Filippo Liviero, Maria Cristina Scarpa, Diego De Stefani, Franco Folino, Manuela Campisi, Paola Mason, Sabino Iliceto, Sofia Pavanello, Piero Maestrelli

**Affiliations:** 1 Department of Cardiac, Thoracic, Vascular Sciences and Public Health, University of Padova, Via Giustiniani 2, 35128 Padua, Italy; 2 Department of Biomedical Sciences, University of Padova, Padua, Italy

**Keywords:** Respiration, Environmental impact, Acute coronary syndromes

## Abstract

A neurogenic pathway, involving airway TRPV-1, has been implicated in acute cardiovascular events occurring after peaks of air pollution. We tested whether inhaled prostaglandin-E_2_ (PGE_2_) and bradykinin (BK) regulate TRPV-1 activity in vivo by changing cough response to capsaicin (CPS) and affecting heart rate variability (HRV), while also taking into account the influence of TRPV-1 polymorphisms (SNPs). Moreover, we assessed the molecular mechanism of TRPV-1 modulation in vitro. Seventeen healthy volunteers inhaled 100 μg PGE_2_, 200 μg BK or diluent in a randomized double-blind fashion. Subsequently, the response to CPS was assessed by cough challenge and the sympathetic activity by HRV, expressed by low (nLF) and high (nHF) normalized frequency components, as well as nLF/nHF ratio. Intracellular [Ca^2+^] was measured in HeLa cells, transfected with wild-type TRPV-1, pre-treated with increasing doses of PGE_2_, BK or diesel exhaust particulate (DEP), after CPS stimulation. Six functional TRPV-1 SNPs were characterized in DNA from each subject. Inhalation of PGE_2_ and BK was associated with significant increases in cough response induced by 30 μM of CPS (cough number after PGE_2_ = 4.20 ± 0.42; *p* < 0.001, and after BK = 3.64 ± 0.37; *p* < 0.01), compared to diluent (2.77 ± 0.29) and in sympathetic activity (nLF/nHF ratio after PGE_2_ = 6.1; *p* < 0.01, and after BK = 4.2; *p* < 0.05), compared to diluent (2.5–3.3). No influence of SNPs was observed on autonomic regulation and cough sensitivity. Unlike PGE_2_ and BK, DEP directly activated TRPV-1. Inhalation of PGE_2_ and BK sensitizes TRPV-1 and is associated with autonomic dysregulation of cardiac rhythm in healthy subjects.

## Introduction

Increments in air pollutants are associated with increased mortality and morbidity due to cardiopulmonary diseases in the hours or few days following exposure, as shown in several epidemiological studies^1,2^. These associations are more consistent for particulate matter (PM), and indeed a worsening of cardiac disease is often observed within a few hours after peaks of PM concentrations in urban air, suggesting that very rapid events take place^3,4^.

The mechanism by which lung exposure to air pollutants is associated with cardiovascular adverse events is currently unclear. Evidence supporting the occurrence of systemic inflammation is inconsistent in the literature and is more compatible with long term rather than acute effects^5^. Alternatively, a neurohumoral mechanism has been hypothesized^6^. Vagal bronchopulmonary receptors such as C-fiber endings are primarily responsible for eliciting centrally mediated reflexes (such as coughing) in response to irritants^7^. There is evidence from animal models and in vitro studies that air pollutants activate sensory Transient Receptor Potential (TRP) channels. Diesel exhaust particulate (DEP) induces cardiovascular adverse effects via activation of TRP Vanilloid 1 (TRPV-1) in rats^6^. Furthermore, TRPV-1 stimulation in rats by inhalation of concentrated ambient particulates causes changes in cardiac rhythm and ECG morphology^8^. Interactions between DEP and TRP Ankyrin 1 (TRPA-1), which are found in airway C-fiber afferents, was also observed by Robinson et al. in guinea pig and human sensory nerves^9^. Therefore, the autonomic control of heart activity seems to be influenced by centrally-mediated reflexes via these afferent unmyelinated C-fibers, which are in turn able to be activated by PM. This may be an explanation for the decrease in heart rate variability (HRV) occurring in susceptible individuals after short-term exposures to PM^10^. This is further supported by our observation that the association between PM exposure and HRV reduction was not detected in patients taking ß-blockers, which regulated their sympathetic activity^11^. However, direct evidence that this mechanism is operative in vivo in humans has never been provided. The function of TRPV-1 in vivo can be investigated with the cough challenge after inhalation of specific agonists, such as capsaicin^12^.

The aim of the present study is to test the hypotheses that the activity of TRPV-1 can be modulated by inhalation of endogenous mediators and that changes in the activity of the TRP channel can interfere with the autonomic regulation of cardiac rhythm. To test these hypotheses in a group of healthy volunteers, we firstly evaluated the cough response to capsaicin before and after inhalation of prostaglandin E_2_ (PGE_2_) and bradykinin (BK), in order to demonstrate sensitization of the TRPV-1 channel in vivo. Secondly, we assessed HRV upon modulation of TRPV-1 function with PGE_2_ and BK. In addition, we assessed whether functional polymorphisms (SNPs) of TRPV-1 can modify the response to PGE_2_ and BK, since capsaicin cough challenge sensitivity in healthy subjects is dependent on multiple TRPV-1 SNPs^13^. Finally, we explored the molecular mechanism of TRPV-1 channel modulation in vitro in HeLa cells transfected with wild type TRPV-1. Overall, we provided direct evidence that modulation of TRP channel activity by inhaled stimuli affects autonomic regulation of HRV in vivo in humans.

## Results

### Cough challenge

Seventeen normal volunteers were recruited: 10 men and 7 women, with a median age of 35 (interquartile range, IQR, 29.5–47). All subjects completed the baseline capsaicin cough challenges without side effects. The Leicester Cough Questionnaire (LCQ) score ranged from 20 to 21, indicating that none of the subjects exhibited cough at baseline, since all the values were nearly at the maximum, i.e. 21. Two subjects were excluded from the study because they felt unwell after inhalation of PGE_2_ (one due to tachycardia, one due to chest tightness).

**Figure 1:**
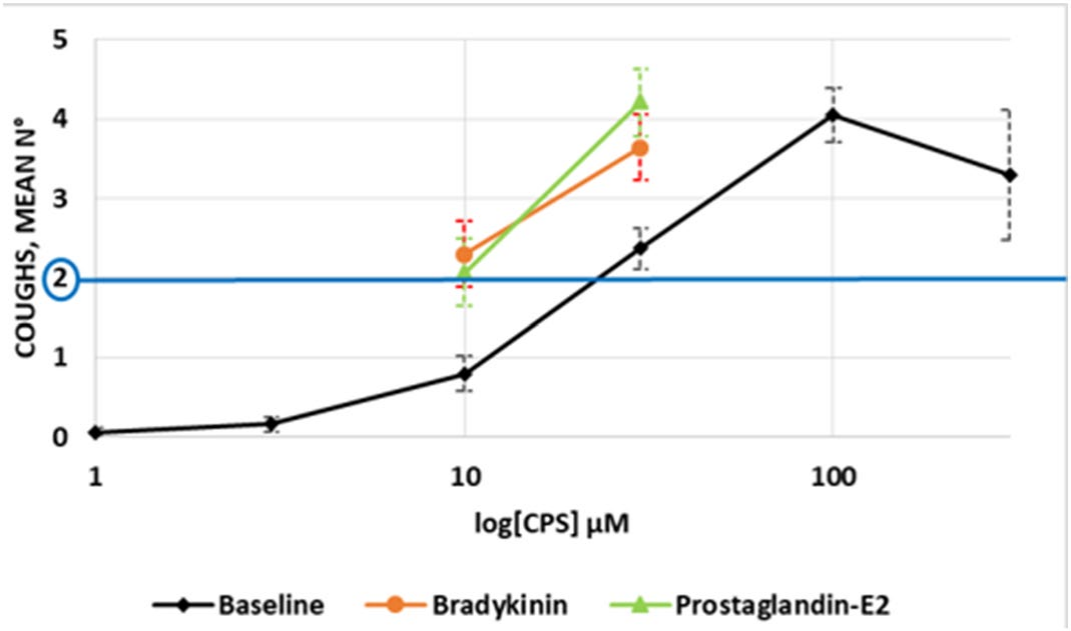
Dose-cough response (mean ± SE) to the cough challenge with capsaicin (CPS). Number of volunteers who completed the tests is 15. Values are displayed as mean ± SE. Black diamonds: full baseline dose-cough response; red circles: cough response to CPS after inhalation of 200 μg bradykinin; green triangles: cough response to CPS after inhalation of 100 μg prostaglandin-E_2_. The line corresponding to 2 coughs indicates the method to calculate the concentration of the agent evoking the two coughs (C2).

The baseline capsaicin dose-cough response curve is displayed in Fig. 1. The median C2 to capsaicin was 23 μM (IQR 12.75–30.0 μM) and the median cough sensitivity, which is expressed as the number of coughs after inhalation of 30 μM of capsaicin, was 2.5 (2.0–3.25). No significant differences in cough response were observed between males and females. The first inhalation of PGE_2_ and BK caused some coughing in a minority of the subjects, while subsequent doses of test mediators were well tolerated without any coughing. The dose-cough responses to CPS after inhalation of PGE_2_ and BK were shifted to the left (Fig. 1).

**Figure 2:**
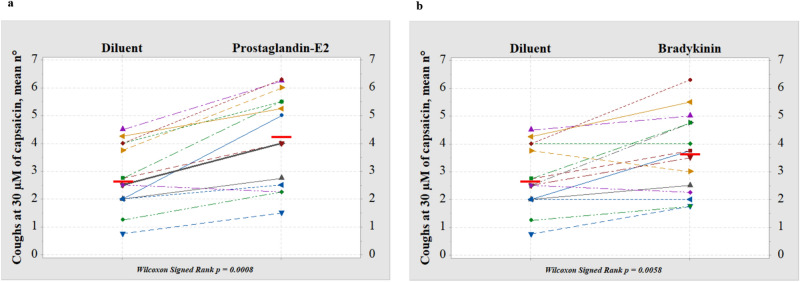
Modulation of the activity of TRPV-1 channels. Number of coughs evoked by 30 μM of capsaicin (n°) after inhalation of prostagliandin-E_2_ (a; n° = 4.20 ± 0.42; *p* = 0.0008) and bradykinin (b; n° = 3.64 ± 0.37; *p* = 0.0058), compared with inhalation of diluent (n° = 2.77 ± 0.29). Horizontal bars represent the mean value. Thicker lines in the plot indicate overlap between two subjects. Wilcoxon Signed Rank was used to assess the changes in cough response before and after inhalation of prostagliandin-E_2_ and bradykinin.

Figure 2 shows the effect of modulation of TRPV-1 with inhaled PGE_2_ (a) and BK (b) in each subject. A consistent increase in the number of coughs in response to 30 μM capsaicin was observed after inhalation of PGE_2_ (*p* = 0.0008) as well as inhalation of BK (*p* = 0.0058) compared with the diluent. Various combinations of four of the six TRPV-1 SNPs with high capsaicin responsiveness (315M, 585I, 469I and 91S) were detected in our subjects (Table 1). There was evidence that the genotype frequencies of two SNPs (rs8065080 and rs222747), deviated from Hardy–Weinberg equilibrium (chi-square test: Χ^2^ = 5.0127 and 12.992, respectively) while no deviation was detected for rs224534 and rs22274 (chi-square test: X^2^ = 0.0194 and 0.4833). Lastly, X^2^ = Not applicable for the remaining two SNPs (rs17633288 and rs9894618). These genetic polymorphisms were unrelated to the modulation of the TRPV-1 channel by PGE_2_ (rho = 0.389, *p* = 0.151) and BK (rho =  − 0.017, *p* = 0.951) as determined by Spearman correlation (see e-Fig. 1a and e-Fig. 1b in additional file 1).

**Table 1:** Distribution of genotype among the participants.

Variable	
Number (M:F)	15 (9:6)
Genotype	Alleles (%)
SNP I585V (rs T8065080C)	
TT	27
CT	60
CC	13
SNP T469I (rs G224534A)	
GG	40
GA	47
AA	13
SNP I315M (rs C222747G)	
CC	47
CG	53
GG	0
SNP P91S (rs C222749T)	
CC	87
TC	13
SNP T505A (rs T17633288A)	
TT	100
SNP K2N (rs 9894618)	
CC	100

### Heart rate variability

Twelve subjects (4 females and 8 males) completed the HRV experiments after modulation of TRPV-1 with PGE_2,_ and 10 subjects (3 females and 7 males) completed the HRV experiments after modulation of TRPV-1 with BK. The effect of aerosol administration was assessed in 8 subjects in the sessions with PGE_2_ and in 7 subjects in the sessions with BK, by comparing HRV before and after inhalation of diluent. The selection of these subjects was performed according to the availability of the subjects to participate to the extra session with the aerosol. Therefore there wasn’t any gender orientation for the selection. The normalized low-frequency power (nLF = LF/TP) represents an index of combined sympathetic and vagal modulation^14^ as well as an index of baroreflex^15,16^, while the normalized HF (nHF = HF/TP) corresponds to an index of vagal modulation. The low/high-frequency power ratio (LHR = LF/HF) is thus the index of sympathovagal balance. None of the HRV parameters exhibited significant changes after inhalation of diluent or significant differences between males and females (data not shown). Table 2 shows that nHF was significantly reduced after activation of TRP channels by inhaled PGE_2_, while nLF was significantly increased (compared with inhalation of diluent). Consequently, the sympathovagal balance expressed by the LF/HF ratio was increased in favor of sympathetic tone. The activation of TRP channels with BK shows the same effects on HRV parameters: PGE_2_ inhalation caused an increase in heart rate (HR), while no significant changes in HR were detected after BK.

**Table 2:** Description of heart rate variability (HRV) parameters (nHF, nLF, LF/HF, mean HR and mean RR) after diluent inhalation compared to inhalation of endogenous TRPV1 modulators (PGE_2_ and BK).

	Diluent	Prostaglandin-E_2_	p*
HF (n.u.)	28.7 (23.2–42.7)	14.1 (11.3–22.6)	0.0022
LF (n.u.)	71.2 (57.3–76.8)	85.9 (77.4–89.0)	0.0022
LF/HF	2.5 (1.4–3.3)	6.1 (3.6–7.9)	0.0022
Mean HR	64 (58–71)	86 (81–90)	0.0022
Mean RR (ms)	964 (844–1,039)	707 (660–772)	0.0022

	Diluent	Bradykinin	p*
HF (n.u.)	24.8 (17.1–42.5)	19.2 (11.6–23.6)	0.0166
LF (n.u.)	75.1 (57.5–82.9)	80.7 (76.4–88.4)	0.0166
LF/HF	3.28 (1.35–4.9)	4.2 (3.2–7.6)	0.0218
Mean HR	67 (62–79)	68 (66–84)	0.066
Mean RR (ms)	897 (755–968)	878 (713–907)	0.046

Value are expressed as median (IQR_25–75_). * Wilcoxon Signed Rank Test.

**Figure 3:**
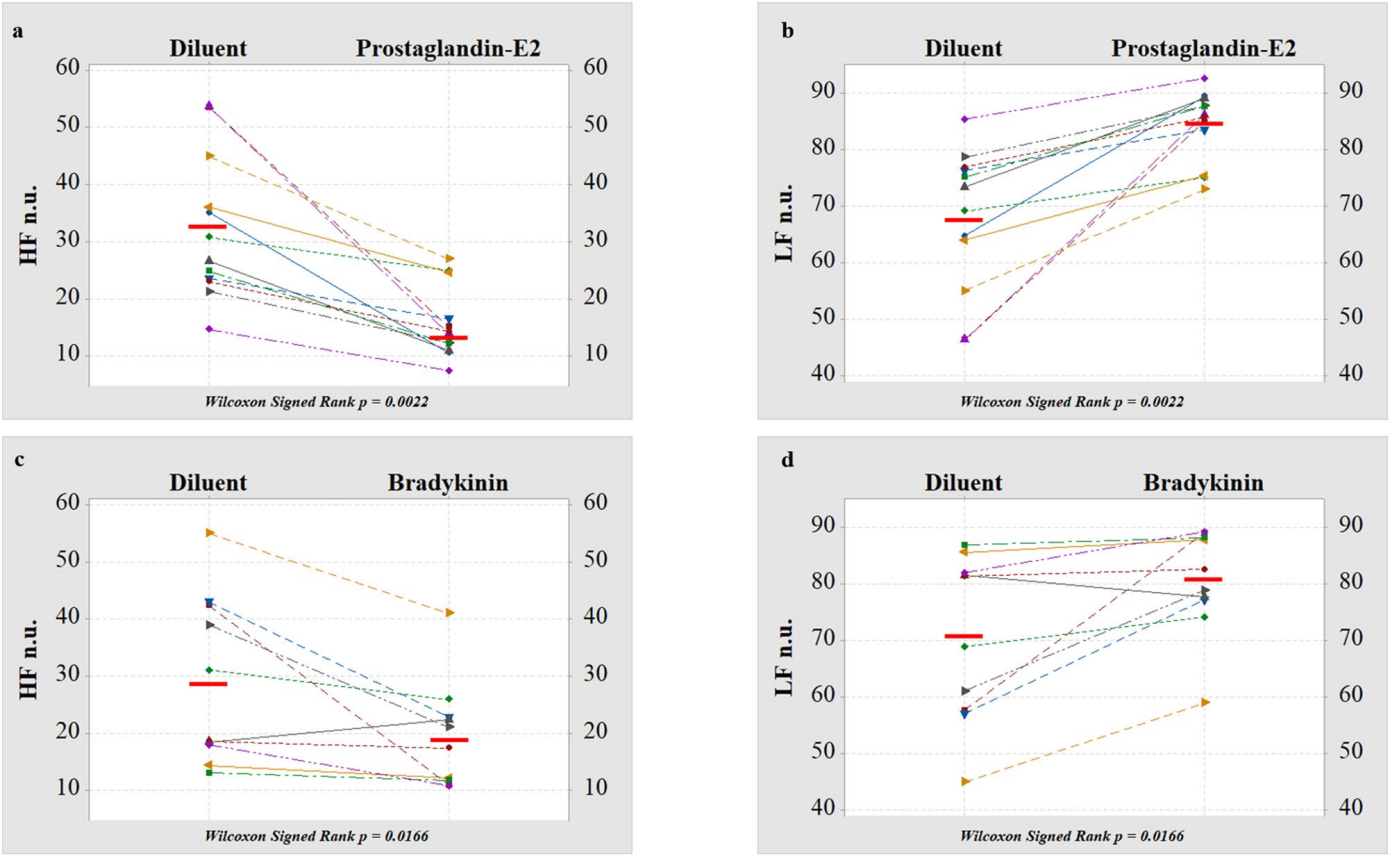
Changes in heart rate variability (HRV) after inhalation of TRP channel modulators,prostagliandin-E_2_ (**a**, **b**) and bradykinin (**c**,**d**), compared with inhalation of diluent, were determined in n = 12 and n = 10 subjects. Normalized high frequency power, nHF (**a**,**c**) and normalized low frequency power, nLF (**b**,**d**) of spectral components in the frequency domain are shown. Horizontal bars represent the mean value. Wilcoxon Signed Rank was used to assess the changes in HRV before and after inhalation of prostagliandin-E_2_ and bradykinin.

Figure 3 shows the individual nHF (a, c) and nLF (b, d) changes compared with diluent. The effect after PGE_2_ (a, b) and BK (c, d) was determined in n = 12 and n = 10 subjects. Overall, the data demonstrated a consistent behavior among individuals. Genetic polymorphisms of the TRPV-1 channel did not influence the HRV in response to PGE_2_ and BK (data not shown).

### *Functionality of TRPV-1 *in vitro

We developed a cell-based assay to test the expression and functionality of the TRPV-1 channel. RNA-seq analysis of ENCODE cell lines revealed that most of the members of the TRP family, including TRPV-1, are not expressed in HeLa cells (see e-Table 1 in additional file 1). Therefore, HeLa cells represent the ideal heterologous system to express the TRPV-1 channel.

**Figure 4:**
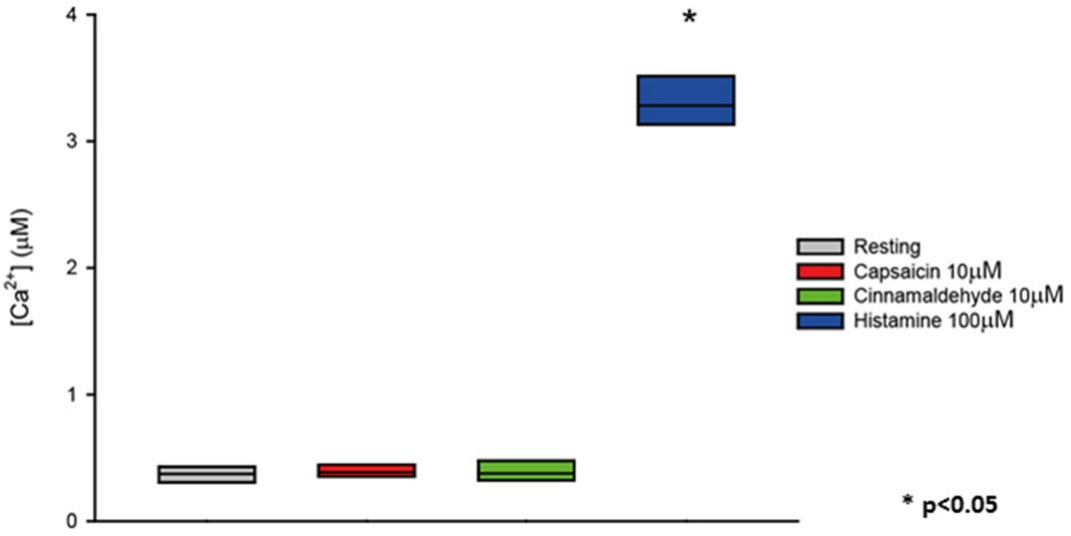
Boxplot representing [Ca^2+^] measurements in control HeLa cells. Where indicated, cells were challenged with 10 μM capsaicin, 10 μM cinnamaldehyde or 100 μM histamine. Variance was calculated by one-way, two-way or three-way ANOVA and multiple comparisons were assessed using the Holm–Sidak post hoc test. In box plots, the boundary of the box closest to zero indicates the 25th percentile, the line within the box marks the median, and the boundary of the box farthest from zero indicates the 75th percentile.

We wanted to confirm the absence of TRP in HeLa cells at the functional level. As is known, Ca^2+^ permeable TRP channels generate changes in the intracellular Ca^2+^ concentration ([Ca^2+^]) through Ca^2+^ influx across the plasma membrane^17^. We took advantage of a widely used genetically-encoded Ca^2+^ indicator based on the photoprotein aequorin^18,19^ to dynamically monitor the selective activation and functionality of TRPV-1 in intact cells. We measured intracellular [Ca^2+^] in response to capsaicin and cinnamaldehyde, which are selective agonists of TRPV-1 and TRPA-1, respectively. As shown in Fig. 4, neither capsaicin nor cinnamaldehyde activate Ca^2+^ signaling in normal HeLa cells, while histamine stimulation induces a transient increase of intracellular [Ca^2+^].

Indeed, HeLa cells are known to express the histamine H1 receptor which, upon activation, triggers the release of Ca^2+^ from intracellular stores through the GPCR-PLC-IP_3_ signaling cascade. Overall, this experiment demonstrates that HeLa cells do not express TRPV-1 and TRPA-1 channels.

**Figure 5:**
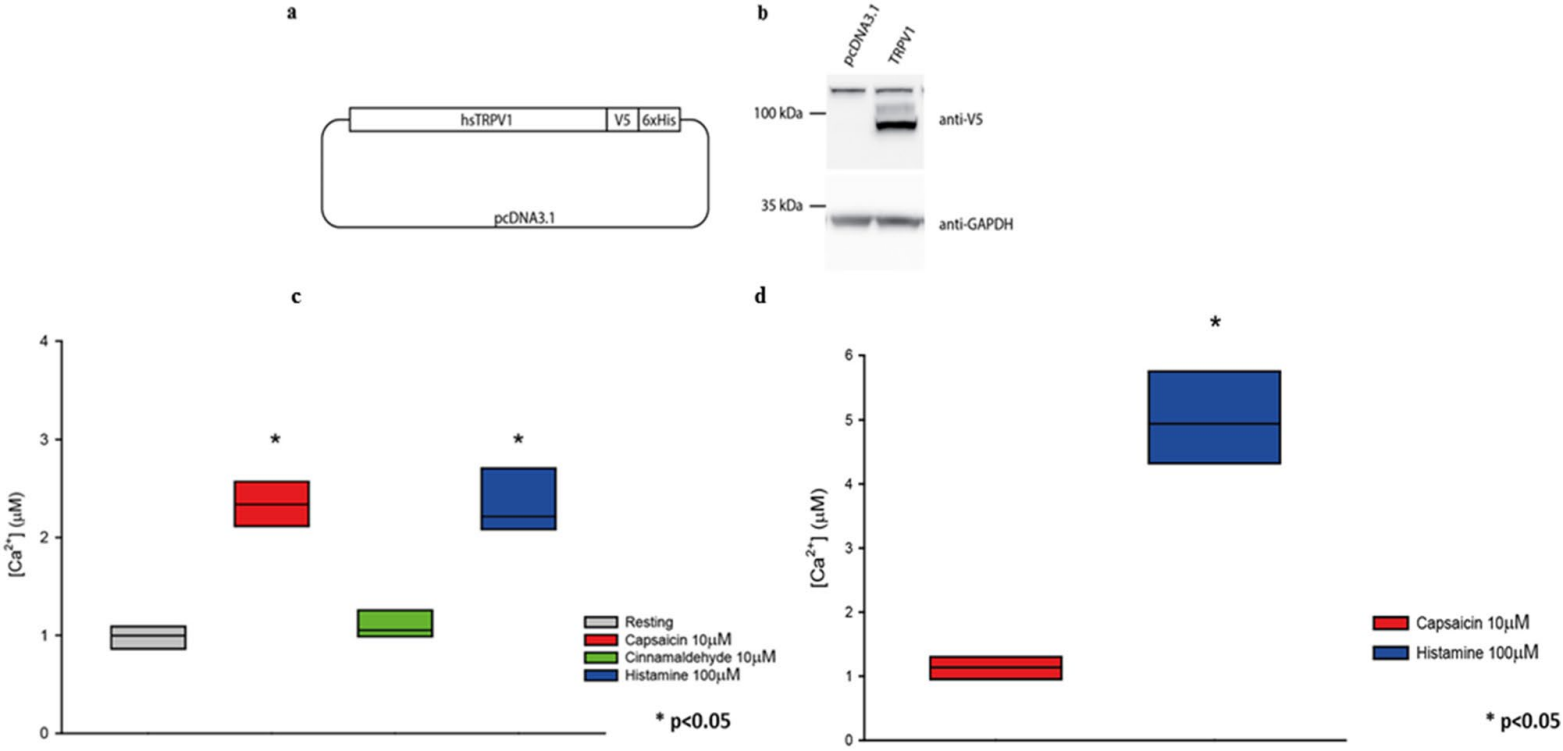
(**a**) Schematic representation of the human TRPV-1 (hsTRPV-1) expressing plasmid. (**b**) HeLa cells were transfected with control pcDNA3.1 or the TRPV-1-encoding plasmid. Total protein was extracted, separated by SDS-PAGE, and a Western blot was performed by probing the membrane with V5 and GAPDH (loading control) antibodies. (**c**) Boxplot representing [Ca^2+^] measurements in HeLa cells expressing TRPV-1. Where indicated, cells were challenged with 10 μM capsaicin, 10 μM cinnamaldehyde or 100 μM histamine. (**d**) Boxplot representing [Ca^2+^] measurements in HeLa cells expressing TRPV-1 perfused with a Ca^2+^-free buffer. Where indicated, cells were challenged with 10 μM capsaicin or 100 μM histamine. Variance was calculated by one-way, two-way or three-way ANOVA and multiple comparisons were assessed using the Holm–Sidak post hoc test. In box plots, the boundary of the box closest to zero indicates the 25th percentile, the line within the box marks the median, and the boundary of the box farthest from zero indicates the 75th percentile.

**Figure 6:**
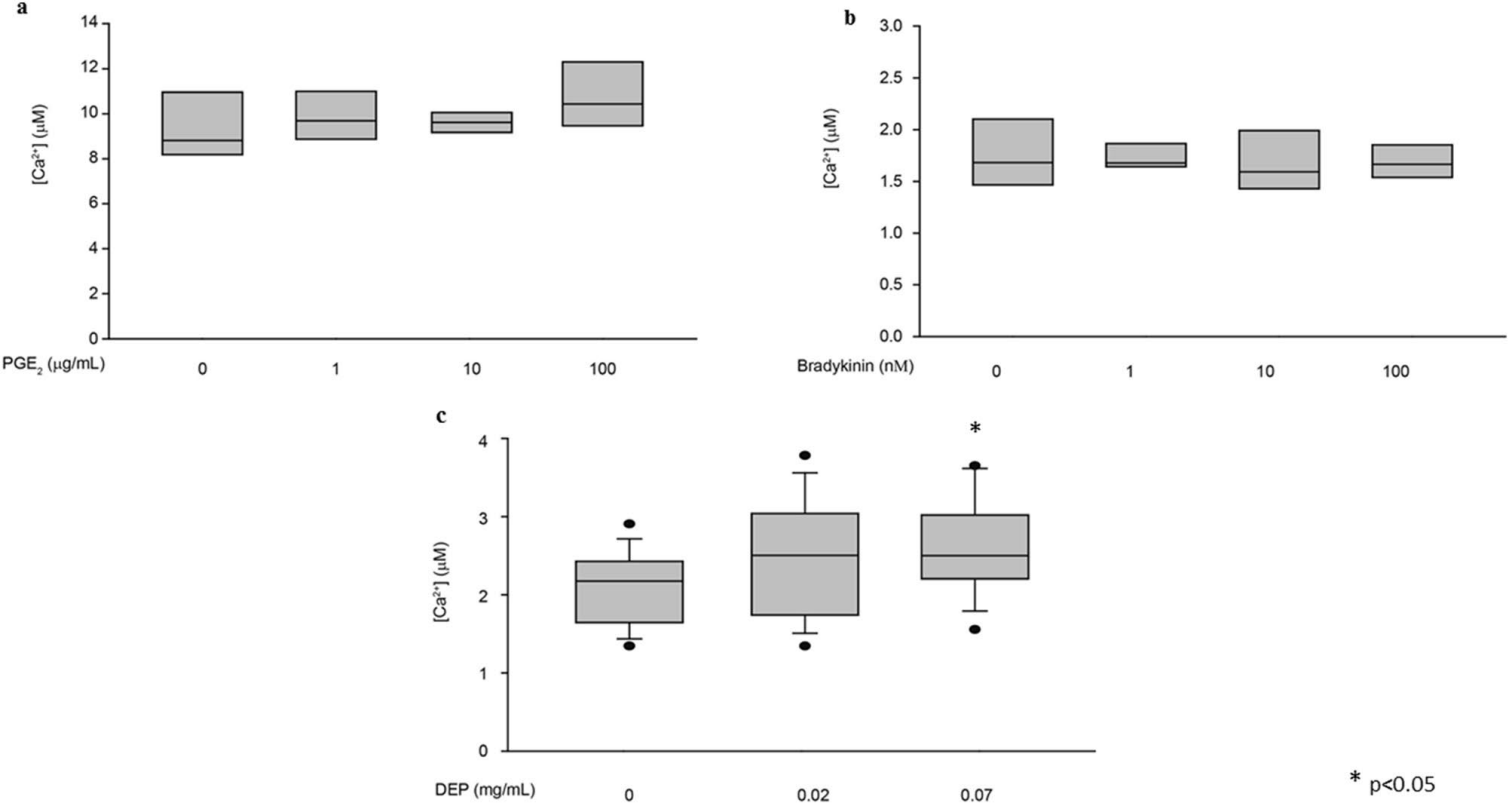
(**a**) Boxplot representing [Ca^2+^] measurements in HeLa cells expressing TRPV-1 and challenged with 100 nM capsaicin (peak values). Cells were pre-treated with the indicated dose of PGE_2_ for 1 h. (**b**) Boxplot representing [Ca^2+^] measurements in HeLa cells expressing TRPV-1 and challenged with 10 nM capsaicin (peak values). Cells were pre-treated with the indicated dose of bradykinin for 1 h. (**c**) Boxplot representing [Ca^2+^] measurements in HeLa cells expressing TRPV-1 and challenged with 10 nM capsaicin (peak values). Cells were pre-treated with the indicated dose of DEP for 1 h. In box plots, the boundary of the box closest to zero indicates the 25th percentile, the line within the box marks the median, and the boundary of the box farthest from zero indicates the 75th percentile. Whiskers (error bars) above and below the box indicate the 95th and 5th percentiles. Dots represent outlying points. Variance was calculated by one-way, two-way or three-way ANOVA and multiple comparisons were assessed using the Holm–Sidak post hoc test.

Next, we transfected HeLa cells with a plasmid encoding the human TRPV-1 channel with a V5-tag fused at the C-terminal (Fig. 5a). After verifying protein expression by Western blot (Fig. 5b), we measured [Ca^2+^] in cells expressing heterologous TRPV-1. Figure 5c shows that addition of capsaicin to TRPV-1-expressing HeLa cells triggers an increase in intracellular [Ca^2+^], as does the positive control histamine. Conversely, cinnamaldehyde is ineffective, causing no significant increase in intracellular [Ca^2+^]. Finally, we wondered whether TRPV-1 mediates Ca^2+^ influx from the extracellular milieu in this system as well. To test this, we stimulated TRPV-1-expressing HeLa cells with CPS during the perfusion of a Ca^2+^-free extracellular buffer. As shown in Fig. 5d, treatment with CPS does not elicit any increase in [Ca^2+^], nor Ca^2+^ influx from the extracellular milieu (which would occur if TRPV-1 were present). Conversely, a Ca^2+^ response is detected with the positive control histamine. Overall, these data confirm that TRPV-1 does not mediate Ca^2+^ influx from the extracellular milieu, and thus that HeLa cells are an ideal heterologous system to study the function of this channel. Finally, we asked whether the presence of concomitant stimuli could have an impact on TRPV-1 functionality. We thus measured TRPV-1 functionality in cells treated with PGE_2_, BK or diesel exhaust particulate matter (DEP). For these experiments, we first pre-treated cells with increasing doses of each agent. Neither PGE_2_ nor BK elicited any increase in [Ca^2+^] on their own, at any of the concentrations investigated. After 1 h, these pre-treated cells were stimulated with capsaicin, and the [Ca^2+^] peak was used as readout of TRPV-1 functionality. Treatment with either PGE_2_ or BK did not modify capsaicin-induced cellular responses (Fig. 6a,b). This suggests that these two inflammatory mediators do not directly sensitize the TRPV-1 channel in our experimental model, possibly because of a lack of a specific transduction pathway. However, treatment with the highest dose of DEP significantly increased TRPV-1-mediated cellular responses (Fig. 6c). These data indicate that TRPV-1 is directly activated by DEP.

## Discussion

In this work, we established that TRPV-1 is sensitized by PGE_2_ and BK inhalation in healthy subjects. The modulation of TRPV-1 activity by these inhaled agents could then influence the autonomic regulation of HRV in our subjects by increasing sympathetic activity and decreasing vagal activity. Based on in vitro experiments, we demonstrated that PGE_2_ and BK act indirectly on TRPV-1 function. In contrast, TRPV-1 activity is directly increased by stimulation with DEP. These results provide the evidence that airway TRPV-1 is implicated in the autonomic regulation of cardiac rhythm in vivo and suggest that peak pollution may induce acute effects on cardiovascular function by a neurogenic mechanism.

The evidence that airway TRPV-1 is sensitized in vivo was obtained using the cough response to capsaicin, which is a specific ligand of TRPV-1. This was done following pre-treatment with one of two independent inhaled stimuli, PGE_2_ or BK, administered at doses that were unable to induce cough in normal subjects. The increase in cough response following capsaicin exposure was consistent among individuals and between sensitizing agents. These results build upon those of Choudry et al.^20^ in which 6 normal volunteers were treated using PGE_2_ or BK, albeit at does that were 3–fourfold higher than in our study. These doses caused coughing in all 6 subjects on their own, but the authors were able to demonstrate a significant effect of PGE_2_, but not of BK, on capsaicin-induced coughing.

In addition to TRPV-1, PGE_2_ and BK were shown to also activate the TRP Ankyrin 1 (TRPA-1) channel in isolated guinea pig sensory nerves^21,22^. TRPA-1 is a target of a broad group of exogenous stimuli including environmental irritants^23^. Based on in vitro and animal models, PGE_2_ and BK are reported to be sensitizers for both TRPV-1 and TRPA-1. The function of TRPA-1 can be assessed in vivo in humans with the cough challenge using inhaled cinnamaldehyde (CNM) as a specific agonist^24^. However, in our healthy volunteers the cough induced by CNM was milder than that induced by CPS, and the dose-cough response to CNM was almost flat. In addition, TRPA-1 was not consistently sensitized by inhaled PGE_2_ or BK, as no significant effect was seen after BK and the signal after PGE_2_ administration was weak (data not shown). Thus, the current study provided evidence of TRPV-1 sensitization after inhalation of these two endogenous mediators in humans in vivo.

Some agonists are thought to be able to both activate and sensitize TRPV-1 channels, but it is difficult to distinguish whether a particular stimulus acts as a direct opener or rather a sensitizer, lowering the activation threshold for another stimulus^25^. PGE_2_ and BK bind to specific G Protein-Coupled Receptors (GPCRs) on the cell membrane (EP3 and B2 receptors respectively) and initiate an intracellular signaling cascade. It has been shown that PGE_2_ activates guinea pig, mouse and human sensory nerves in vitro and causes coughing in animal models via EP3 receptor activation^26^. In guinea pigs, BK activates sensory nerves and elicits coughing via activation of B2 receptors^21^. TRPV-1 is also thought to play a role in some GPCR signaling pathways, for example those implicated in BK and PGE_2_ activity. Indeed, TRPV-1-selective antagonists partially inhibit the tussive response to PGE_2_ and BK in a guinea pig cough model^21^. The pathways downstream of GPCR coupling that lead to either sensitization or activation of TRPV-1 have not been fully elucidated, but probably involve the production of diacylglycerol (DAG) and the activation of phosphokinase C (PKC), as DAG and PKC have been found to directly bind the TRPV-1 channel^27^. It was unknown whether TRPV-1 can also be activated by PGE_2_ and BK in the absence of the above GPCR-mediated pathways. Our experiments in vitro demonstrated that PGE_2_ and BK are unable to open functional TRPV-1 channels expressed by HeLa cells, which lack EP3 and B2 receptors. Therefore, these endogenous mediators could be considered to be indirect sensitizers of TRPV-1. This may be one of the reasons why the effect of PGE_2_ and BK on the channel is irrespective of the presence of TRPV-1 SNPs. In contrast, particulate matter in air pollution, such as DEP, directly interacts with TRPV-1 and causes channel opening. The DEP molecular components responsible for the effect on TRPV-1 have not yet been determined. While the soluble organic material embedded on the particulate carbon core of DEP is a candidate for TRPA-1 activation^9^, TRPV-1 was also shown to be activated by the solid components of coal fly ash particulate matter^28^. The size of the DEPs studied were in the range of the respirable fraction which is expected to deposit in the respiratory tract when inhaled in vivo, and which also corresponds to the particle size that is most effective in TRP channel activation in vitro^29^. It is difficult to establish whether the amount of DEP used for the in vitro experiments is comparable to that which is inhaled in an area with poor air quality due to vehicle traffic. However, the concentrations of DEP used are similar to those used in previous in vitro cell stimulation investigations^9,28,30^.

Activation of airway sensory nerves by air pollutants in animal models causes an imbalance of autonomic efferent pathways that are mediated centrally at the level of the mid-brain. This produces cardiovascular function changes including an increase in systolic blood pressure, alteration of cardiac rhythm and alteration of electrocardiogram morphology^6,8,31^. To date there are no studies on the functional changes of TRPV-1 after DEP stimulation in humans. There is in humans, a short term experimental exposure of healthy subjects to diesel exhaust fumes that causes impairment of HRV occurring at the end of the 2 h exposure session^32^. Although these findings provide some insights into the interaction between inhaled pollutants and acute cardiovascular effects in man, the mechanism remains undetermined. We have observed that an increase in sympathetic activity can be induced by stimuli that are also able to sensitize airway TRPV-1. This provides evidence that signals from airway sensory nerves, once they are integrated at the CNS level, can influence autonomic drive to the heart, as was previously shown in animal models^6,8,31^. Posture and breathing may also influence HRV, but we can reasonably exclude any confounding effect of these variables. During experiments, ECG recording was performed at rest in a supine position. Respiratory frequency was not controlled but is very unlikely to have been out of the HF band, i.e. slow breathing < 0.15 Hz (9 breath/min) or fast breathing > 0.4 Hz (24 breath/min)^33^. The normalization of the LF and HF data by TP minimized the effect of changes in heart rate on spectral components of HRV^34^. In addition, the changes in HRV after TRPV-1 sensitization appear independent on the changes in HR, since they were observed after inhalation of BK which, in contrast to PGE_2_, does not induce tachycardia. Sensitization of TRPV-1 was also associated with an increase in sympathetic activity and a concomitant reduction of parasympathetic drive. Therefore, these changes in autonomic activity may be a common pathway leading to increased morbidity and mortality from various conditions, including cardiovascular disease. Indeed, substantial evidence exists to support the notion that decreased HRV precedes the development of a number of cardiovascular disease risk factors^35^.

Our findings identified a mechanism, which is functional in vivo in humans, by which sensitization of airway sensory TRPV-1 channels by inhaled agents induces an increase in sympathetic activity. Since there is evidence that air pollutants can activate TRP channels, we provide a proof of concept that may explain why exposure to peaks of pollutants is associated with short-term cardiovascular adverse events.

## Methods

### Subjects

Seventeen healthy volunteers were recruited. An approximately equal number of males and females were included. All patients were required to be non-smokers, free of acute or chronic diseases according to medical history and physical examinations, and not taking any regular medication. The study was approved by the local Research Ethics Committee (4057/AO/17) and all subjects gave written informed consent. All methods were performed in accordance with the relevant guidelines and regulations.

### Study design

Leicester Cough Questionnaires (LCQ) were administered before the cough challenges and the scores were evaluated in order to exclude subjects with cough symptoms^36^. The subjects were required to abstain from ingesting spicy food, garlic, cinnamon and from carrying out outdoor sporting activities within 24 h prior to the tests.

Subjects attended the laboratory on six separate occasions (V1–V6). Baseline cough responses to increasing dilutions of inhaled CPS were assessed (V1). Endogenous modulators of TRPV-1 (PGE_2_ and BK) or diluent were inhaled in a random order, double-blind fashion, and the cough challenge with CPS was repeated immediately after the last breath of the test modulator (V2-V4). HRV was assessed in each subject after diluent and PGE_2_ or diluent and BK, which were inhaled at the same concentration and in the same way as for the cough challenge experiments (V5-V6). The experiments were conducted at intervals of at least 5 days.

The presence of six nonsynonymous functional polymorphisms of TRPV-1 (K2N rs9894618, P91S rs222749, I315M rs222747, T469I rs224534, T505A rs17633288 and I585V rs8065080) was assessed in blood DNA samples collected from each subject at V1.

In order to determine whether PGE_2_ and BK have a direct effect on TRPV-1 activity, functional studies on HeLa cells transfected with the wild type TRPV-1 channel were performed in vitro using capsaicin as an agonist.

### CPS cough challenge

The cough challenge was performed according to current guidelines^12^. Briefly, single vital-capacity breaths of incremental concentrations of CPS (1–1,000 µM; Sigma-Aldrich S.r.l., Milan, IT) were inhaled at 2-min intervals from a nebulizer controlled by a dosimeter (Mefar MB3 CE, Mefar S.p.A, Brescia, Italy). Inhalation flow was limited to approximately 0.5 L/s by applying a critical orifice to the inlet of the nebulizer. Each concentration of the tussive agent was inhaled four times with a 30-s pause between each inhalation. Both the subjects and the investigator were blind to the concentrations of the tussive agent. The number of coughs in the 15-s following each inhalation was recorded using Digital Audio Tape recorder and counted. The average number of coughs after each concentration of the tussive agent was calculated and a concentration response curve for each test was constructed. The concentration of the agent evoking at least two coughs (C2) was determined.

### Modulation of TRPV-1

PGE_2_ and BK were used as modulators of TRPV-1 since there is evidence that they activate the TRP channel expressed by human sensory nerves in vitro^21^. The PGE_2_ stock solution was prepared by diluting 10 mg of PGE_2_ (Tocris Bioscience, Bristol, U.K.) with 1 mL dehydrated alcohol. The resulting 10 mg/mL solution was aliquoted into 0.1 mL single-use vials and stored at − 70 °C. The BK (Sigma-Aldrich S.r.l., Milan, Italy) stock solution was prepared by diluting 20 mg of BK in 1 mL of 10% ethanol in 0.9% NaCl. The resulting 20 mg/mL solution was aliquoted into 0.1 mL single-use vials and stored at − 70 °C. Prior to use the stock solutions were diluted with 0.8 mL 0.9% NaCl solution. The solutions of diluent, PGE_2_ and BK were inhaled via a De Vilbiss 646 nebulizer connected to a dosimeter (Mefar MB3) with an output of 18 μl per breath. The subjects inhaled five breaths of each solution resulting in a total administration of 100 μg of PGE_2_ and 200 μg of BK. These doses were chosen since they exhibited biological effects in humans by inhalation and can be safely delivered by aerosol^37,38^.

### Assessment of HRV

HRV was assessed by ECG obtained in a supine position and using a device connected to the patient via two electrodes. HRV data were acquired by a Bluetooth acquisition system (BT16 Plus, FM, Monza, Italy). ECG was recorded at rest for at least 5 min. Then, each subject inhaled 5 breaths of nebulized diluent from a dosimeter and ECG was recorded for at least 5 min. Subsequently, the subject inhaled 5 breaths of PGE_2_ or BK, in random order and on different days, at the same concentrations used in the modulation of cough response.

HRV was analyzed using Kubios HRV software (ver. 3.3)^39^. Normal and aberrant complexes were identified and all of the adjacent intervals between normal beats over 5 min intervals were considered. We analyzed the spectral components (HRV frequency domain variables) as the absolute values of power (ms^2^)^18^. Power spectral density was analyzed by Fast Fourier Transform. The main spectral components were very low frequency (VLF), low frequency (LF), high-frequency (HF) and the LF/HF ratio. The area under the curve of the spectral peaks within the frequencies 0.01–0.4, 0.01–0.04, 0.04–0.15, and 0.15–0.40 Hz were defined as the total power (TP), very low-frequency power (VLF), low-frequency power (LF), and high-frequency power (HF), respectively. In order to normalize LF and HF, we used the total power within the frequency range of 0.01–0.4 Hz.

### Genotyping of TRPV-1 SNPs

Six nonsynonymous functional polymorphisms of TRPV-1 (K2N rs9894618, P91S rs222749, I315M rs222747, T469I rs224534, T505A rs17633288 and I585V rs8065080) were evaluated in DNA extracted from blood samples as previously described^13^. Briefly, genotyping was performed by commercially available KASP Assay mix (containing the target specific primers) and KASP Master mix. Reactions were set up according to the manufacturer's protocol. As previously described^13^, samples were run in triplicate on a Steponeplus Real-Time instrument (Applied Biosystems, Foster City, CA) and amplification conditions were equivalent (first step at 94 °C for 15 min, then 10 cycles of 94 °C for 20 s and 61–55° for 60 s (dropping by 0.6 °C per cycle), 26 cycles of 94 °C for 20 s and 55 °C for 60 s). Allelic discrimination was performed using the SDS software v2.3 (Applied Biosystems).

### Chemicals, cell culture and transfection

As previously described^40^, all chemicals were purchased from Sigma-Aldrich, unless otherwise specified. All the experiments were performed in HeLa cells (ATCC Number: CCL-2) cultured in Dulbecco’s modified Eagle’s medium (DMEM) (Gibco #41966052, Thermo Fisher Scientific, Milan, Italy), supplemented with 10% fetal bovine serum (FBS) (Thermo Fisher Scientific), containing penicillin (100 U/ml) and streptomycin (100 μg/ml) (Euroclone). Prior to experimentation, cells were seeded onto 13-mm diameter glass coverslips (no. 1.5) and allowed to grow to 50% confluence before transfection. Transfection was performed with a standard Ca^2+^-phosphate procedure. The TRPV-1-expression plasmid was a kind gift from Christopher A. Reilly^40^, and the mock vector pcDNA3.1 (Thermo Fisher Scientific) was used as control. Diesel exhaust particulate DEP (Diesel particulate matter, Standard Reference Material 2975) was purchased from the National Institute of Standards and Technology, Boulder, CO, USA. The particle size distribution of DEP suspensions was characterized using a laser analyzer (DIPA 2000, Donner Technologies Ltd, Or Aquiva, Israel) before and after sonication for 10 min. The median diameter of the particles was 0.9 μm (range 0.2–6 μm) and the diameter distribution did not change after sonication, indicating that the particles did not aggregate in the experimental conditions.

### [Ca^2+^]_mt_ measurements

HeLa cells were grown on 13 mm diameter round glass coverslips to 50% confluence and cotransfected with a cytosolic aequorin-based probe (cytAEQ) together with the indicated plasmids. Thirty-six or forty-eight hours after transfection, cells were incubated with 5 μM coelenterazine for 1–2 h in KRB (Krebs–Ringer modified buffer: 125 mM NaCl, 5 mM KCl, 1 mM Na_3_PO_4_, 1 mM MgSO_4_, 5.5 mM glucose, 20 mM HEPES, pH 7.4) at 37 °C supplemented with 1 mM CaCl_2_, and then transferred to the perfusion chamber. All aequorin measurements were carried out in KRB. For experiments performed in Ca^2+^-free conditions, KRB was supplemented with 100 μM EGTA. Agonists and other drugs were added as specified in the text. The experiments were terminated by lysing cells with 100 μM digitonin in a hypotonic Ca^2+^-rich solution (10 mM CaCl_2_ in H_2_O), thus discharging the remaining aequorin pool. The light signal was collected and calibrated into [Ca^2+^] values by an algorithm based on the Ca^2+^ response curve of aequorin at physiological conditions of pH, [Mg^2+^], and ionic strength, as previously described^41^. Alternatively, [Ca^2+^] measurements were carried out on a PerkinElmer Envision plate reader equipped with a two-injector unit. Cells were transfected as described above in 24-well plates and then re-plated into 96-well plates (1:5 dilution) the day before the experiment. After reconstitution with 5 μM coelenterazine, cells were placed in 70 μl of KRB and the luminescence from each well was measured for 1 min. During the experiment, histamine was first injected at the desired concentration to activate calcium transients, and then a hypotonic, Ca^2+^-rich, digitonin-containing solution was added to discharge the remaining aequorin pool. Output data were analyzed and calibrated with a custom made macro-enabled Excel workbook. Functional TRPV-1 data were representative of at least three independent transfections.

### Statistical analysis

Nonparametric statistics were used to assess the changes in cough response to capsaicin after TRPV-1 stimulation with PGE_2_ and BK (Wilcoxon Signed Rank). Wilcoxon Signed Rank was also used to test the difference in ECG parameters between diluent, and PGE_2_ or BK. Variance was calculated by one-way, two-way or three-way ANOVA as indicated in the legends, and multiple comparisons were assessed using the Holm-Sidak post hoc test.

The relationship of the genetic polymorphisms with the modulation of the TRPV-1 channel by PGE_2_ and BK was determined by Spearman correlation. The statistical analyses were performed using StatView, Minitab and SigmaPlot. A *p* value of < 0.05 was considered statistically significant.

### Ethics approval and consent to participate

The study was approved by the local Research Ethics Committee (4057/AO/17) and all subjects gave written informed consent.

## Supplementary information

Supplementary Information 1

## Data Availability

All data is available upon request.

## Abbreviations

BK: Bradykinin
CPS: Capsaicin
C2: The concentration of capsaicin causing 2 coughs
DEP: Diesel exhaust particulate
HRV: Heart rate variability
LCQ: Leicester Cough Questionnaire
nHF: High normalized frequency components
nLF: Low normalized frequency components
SNPs: Single nucleotide polymorphisms
PGE2: Prostaglandin-E2
TRPA-1: Transient receptor potential ankyrin 1
TRPV-1: Transient receptor potential vanilloid-1
