# Supplementary material for: Modulation of TRPV-1 by prostaglandin-E2 and bradykinin changes cough sensitivity and autonomic regulation of cardiac rhythm in healthy subjects
Source: Sci Rep. 2020 Sep 16;10:15163. doi: 10.1038/s41598-020-72062-y (PMC7494872; doi:10.1038/s41598-020-72062-y)
Supplement: Supplementary file 1 — Supplementary Information 1 [file 41598_2020_72062_MOESM1_ESM.doc]

**Supplementary materials**

**Modulation of TRPV-1 by prostaglandin-E_2_ and bradykinin changes cough sensitivity and autonomic regulation of cardiac rhythm in healthy subjects**

Filippo Liviero MD, PhD^1^, Maria Cristina Scarpa PhD^1^, Diego De Stefani PhD^2^, Franco Folino MD^1^, Manuela Campisi MSc^1^, Paola Mason MD, PhD^1^, Sabino Iliceto MD^1^, Sofia Pavanello PhD^1^**^*^** and Piero Maestrelli MD^1^

^1^Department of Cardiac, Thoracic, Vascular Sciences and Public Health, University of Padova, Italy.

^2^Department of Biomedical Sciences, University of Padova

***Corresponding Author**

Prof. Sofia Pavanello

Department of Cardiac, Thoracic, Vascular Sciences and Public Health, University of Padova

Via Giustiniani 2, 35128 Padova, Italy

e-mail: sofia.pavanello@unipd.it

**Keywords: TRP channels, Diesel exhaust particulate (DEP), heart rate variability, SNPs, cough challenge; inhalation**

**Summary conflict of interest statements:** the authors have reported that no potential conflicts of interest exist with any companies/organizations whose products or services may be discussed in this article.

#### e-Table 1 Expression of Transient Receptor Potential (TRP) channels in various cell lines. Data are reported as Transcripts Per Kilobase Million of RNA-seq of long poly adenylated RNA and long non poly adenylated RNA from ENCODE cell lines (dataset E-GEOD-26284). None of the TRP channels is expressed by HeLa cell line.

| **Gene** | **A549** | **AG445** | **BJ** | **GM12878** | **HUVEC** | **HeLa** | **HepG2** | **K562** | **MCF-7** | **NHLF** | **SK-N-SH** |
| --- | --- | --- | --- | --- | --- | --- | --- | --- | --- | --- | --- |
| **TRPV1** |  |  |  |  |  |  |  |  |  |  |  |
| **TRPA1** | **2** | **36** |  |  |  |  |  |  |  | **36** |  |
| **TRPV-4** |  | **2** | **5** |  | **13** |  | **3** |  | **2** |  | **3** |
| **TRPM-4** |  |  | **3** |  | **6** |  |  | **3** | **3** |  | **6** |
| **TRPC-4** |  |  | **4** |  |  |  |  |  |  | **7** |  |
| **TRPC-6** |  | **2** | **3** |  |  |  |  |  |  | **7** |  |
| **TRPM-2** |  |  |  |  |  |  |  |  |  |  |  |
| **TRPC-1** | **9** | **4** | **2** | **2** |  |  | **5** |  | **5** | **5** | **5** |
| **TRPV-3** |  |  |  | **2** |  |  |  |  |  |  |  |
| **TRPV-2** |  | **12** | **2** | **20** | **2** |  | **12** | **17** |  | **26** | **4** |

#### Relationship between individual SNP-based levels of capsaicin responsiveness and Delta cough, that represents the modulation of the TRPV-1 channel with PGE_2_ (e-figure 1a) and BK (e-figure 1b). SNP-based capsaicin responsiveness indicate the incidence (from 0 to 8) of the number of capsaicin-responsive SNPs detected on one or both alleles. Levels of CPS responsiveness ranged between 2 with lowest and 6 the highest levels of responsiveness as previously described [1]. Delta cough was calculated as the difference between the number of coughs obtained at 30 µM of capsaicin, after modulation of TRPV-1 with PGE_2_ (e-figure 1a) and BK (e-figure 1b) compared to diluent. Triangles in the scatterplots indicate overlap between two subjects. Non parametric Spearman linear regression was performed to compare Delta cough of each subject in relation to the number SNP-based levels of capsaicin responsiveness.

**e-Figure 1a**

*
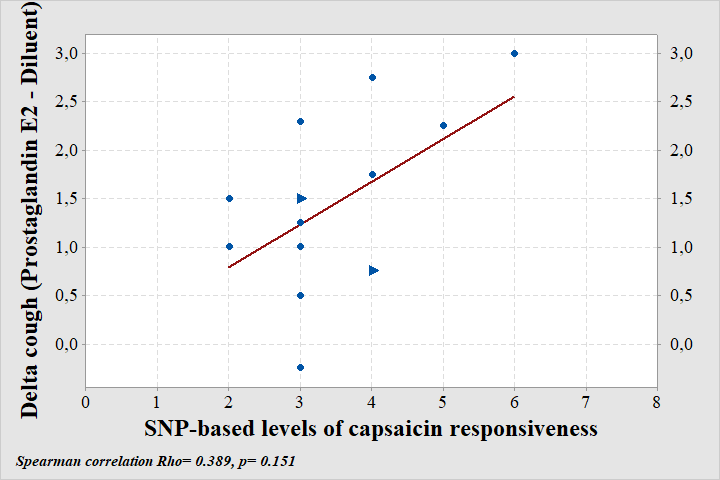
*

**e-Figure 1b**


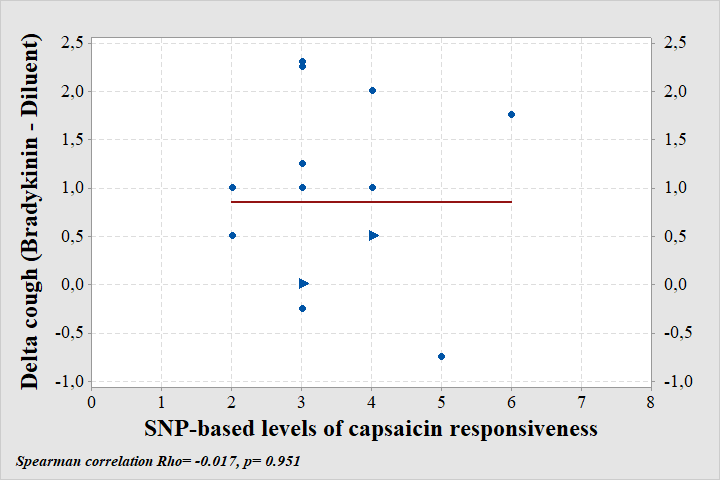


Original and unprocessed versions of blot used in figure 5

**Reference**

1. Liviero, F. *et al.* Multiple single nucleotide polymorphisms of the transient receptor potential vanilloid 1 (TRPV-1) genes associate with cough sensitivity to capsaicin in healthy subjects. *Pulmonary Pharmacology & Therapeutics.* **61,** 1-5 (2020). <https://doi.org/10.1016/j.pupt.2020.101889>.
